# Precrop-treated soil influences wheat (*Triticum aestivum* L.) root system architecture and its response to drought

**DOI:** 10.3389/fpls.2024.1389593

**Published:** 2024-06-04

**Authors:** Jonathan E. Cope, Fede Berckx, Anna Galinski, Jonas Lentz, Kerstin A. Nagel, Fabio Fiorani, Martin Weih

**Affiliations:** ^1^ Department of Crop Production Ecology, Swedish University of Agricultural Sciences, Uppsala, Sweden; ^2^ Institute of Bio- and Geosciences, Plant Sciences (IBG-2), Forschungszentrum Jülich GmbH, Jülich, Germany

**Keywords:** root system architecture, *Triticum aestivum*, precrop effect, water stress, rhizotron, G×E interaction

## Abstract

**Aims:**

Root system architecture (RSA) plays an important role in the plant’s ability to sustain yield under abiotic stresses such as drought. Preceding crops (precrops) can affect the yield of the proceeding crop, partially by affecting the RSA. This experiment aims to explore the interactions between precrop identity, crop genotype and drought at early growth stages.

**Methods:**

Rhizotrons, sized 60 × 80 × 3.5 cm, were used to assess the early root growth of two winter wheat (Triticum aestivum L.) genotypes, using precrop-treated soil around the seedlings and differing water regimes. The rhizotrons were automatically imaged 3 times a week to track root development.

**Results:**

Precrop-treated soil affected the RSA and changes caused by the reduced water treatment (RWT) were different depending on the precrop. Largest of these was the 36% reduction in root depth after wheat, but 44% after OSR. This indicates that effects caused by the precrop can be simulated, at least partially, by transferring precrop-treated soils to controlled environments. The genotypes had differential RSA and reacted differently to the RWT, with Julius maintaining an 8.8-13.1% deeper root system compared to Brons in the RWT. In addition, the combined environmental treatment affected the genotypes differently.

**Conclusion:**

Our results could help explain discrepancies found from using precrops to enhance yield as they indicate differences in the preceding crop effect when experiencing drought stress. Further, these differences are affected by genotypic interactions, which can be used to select and adapt crop genotypes for specific crop rotations, depending on the year. Additionally, we have shown a viable method of stimulating a partial precrop effect at the seedling stage in a controlled greenhouse setting using field soil around the germinated seed.

## 1 Introduction

Wheat is a globally important crop, accounting for 18% of total dietary calories worldwide (Erenstein et al., 2022). In the EU-27 it is one of the most important crops, accounting for the highest production (tonnage) and land use of any primary crop, using 29% of the arable land used for primary crop production (FAOSTAT, 2022).

Climate change is set to impact the production of wheat, both within the EU-27 and worldwide (Ortiz et al., 2008), causing reductions in food security and economic damages. Various methods for “climate-proofing” crops, such as wheat, against the known and unknown factors resulting from climate change have been proposed (Kusunose et al., 2022), including developing root system architecture that promotes resilience in certain environments (Lynch, 2022).

Root system architecture (RSA) is the collective term for the measurements used to observe the structural features of the root system. It includes measurements such as root length, number, angle or spread, and distribution along the soil profile. The plastic nature of the root system and its development allows for plant adaptation to environmental changes (Lynch, 2022). Root classes can be split into multiple distinctions. One such classification, important in the early stages of cereal growth, is between axial and lateral roots. Axial roots are the roots which originate from embryo or shoot tissue, and lateral roots are those that branch off from the axial roots (Dowd et al., 2019).

Development of the RSA during the seedling stage has been linked with important adaptations to genotype-environment (G×E) interactions, such as the reaction to abiotic stress and nutrient use efficiency (Lynch, 2022). The soil structure also affects early RSA, as Mawodza et al. (2021) showed that finer macro-aggregates increase the total lateral root length, whilst coarser macro-aggregates increase axial root growth.

The soil structure and composition, and thus the G×E interactions, can also be influenced by the preceding crop (precrop); affecting factors such as the soil structure (e.g. biopores) (Han et al., 2016), weed and pathogen burden (Köpke and Nemecek, 2010), resource availability (Han et al., 2021), and microbial community (Friberg et al., 2019). The understanding of the precrop effect on the RSA is limited, with most studies assessing the effect of the structural changes to the soil, such as biopores (Han et al., 2016). However, other factors, such as nutrients and microbiome, have been shown to influence the RSA (Giehl et al., 2013; Verma et al., 2021).

In any G×E interaction, abiotic stress can form a large part of the environmental effect. The major abiotic stress tested concerning the RSA is drought stress, with RSA involved in stress mitigation. RSA has been shown to adapt a crop to an environment with abiotic stresses, including drought, through tolerance and tropism – both positive and negative, depending on the stimuli (Karlova et al., 2021). Drought stress often causes parsimonious architectural phenotype, i.e. a reduction in axial and lateral root number, along with a deeper root system depth (Zhan et al., 2015). This type of architecture allows for greater access to deeper resources of both water and nitrogen, however, it reduces the plasticity of the system by reducing the number of roots not tailored for water capture (Lynch, 2022). Water resource use efficiency is additionally linked to the precrop, with the choice of precrop affecting the yield (Sieling and Christen, 2015), and increased diversity in long-term crop rotations having a positive influence on drought resilience (Renwick et al., 2021) reducing grain yield losses (Costa et al., 2024).

The assessment of the RSA traits can be very difficult due to the reduced accessibility to the root, causing limitations of phenotyping below ground. In-field measurements of the root system tend to be destructive (Trachsel et al., 2011), and thus can only be undertaken at one time-point for any individual plant and are often labour-intensive or capture only a section of the RSA. Vessel experiments are a controlled method of RSA analysis, with one method being the rhizotron which allows observation of the root system by using a transparent plate mounted on a soil-filled box. This method was established in the 19^th^ century (Sachs, 1873) and has been expanded upon to create laboratories which monitor multiple boxes (Huck and Taylor, 1982). In a few labs the capture of the RSA is automated, with imaging of the root and shoot growth through cameras and automated machines (Nagel et al., 2012; Liu et al., 2021). This allows for high throughput phenotyping of the root system through early development.

However, a rhizotron phenotyping facility, such as the GrowScreen-Rhizo 1 facility at Forschungszentrum Jülich GmbH (Nagel et al., 2012), has not been used in understanding the effect of the preceding crop on the RSA. Here we assessed the viability of testing the precrop effect in a controlled environment using translocated field soils. This study aimed to assess the effects of precrop-treated soil, as a surrogate to simulate a precrop effect, on early root development, and how this changes in the face of water stress. We explored the hypotheses that the precrop-treated soil affects the early stages of root development, resulting in differences in RSA. Due to differences in starting conditions, such as nutrients, we expected the wheat-treated soil to have a more limiting effect on root growth than the winter oilseed rape (OSR) treated soil (H1); the precrop-treated soil affects the changes in RSA caused by water stress, as the roots will have adapted differently making them more or less prone to drought stress (H2); and the effect of genotype will cause differences in the extent of these changes, as different genetics will result in differences in trait expression (H3).

## 2 Materials and methods

### 2.1 Field soil collection

Soil samples were collected, using a mechanical digger to an approximate depth of 30 cm, from two neighbouring fields in Bjertorp (Västergötland, Sweden) in September 2022. Both fields had recently been harvested, one field had winter wheat harvested from it (wheat precrop-treated soil), and the other had winter oilseed rape (OSR) harvested from it (OSR precrop-treated soil). This soil was processed by breaking up the large sections by spade and then processing it through a mechanical sieve shaker to separate it into different components, soil pieces bigger than 10 mm were then broken down further by spade and re-sieved. The resulting processed soil was then kept in the dark, and covered, for approximately one month. Samples of the soils had their moisture content measured by recording the weights before and after heating in a drying oven (in falcon tubes) at 65°C for 14 days. The moisture content was 9.0% for wheat precrop-treated soil, and 12.4% for the OSR precrop-treated soil.

### 2.2 Phenotyping using rhizotrons

The experiment was run in the GrowScreen-Rhizo 1 facility at Forschungszentrum Jülich GmbH (Nagel et al., 2012). This system uses 72 rhizotrons which have internal dimensions of 60 × 80 × 3.5 cm, with one of the larger sides being made from clear plastic, with an internal volume of approximately 16.8 L. Each box had an approximate 7 cm layer of floral foam lining the bottom (**Supplementary Figure 1**). These rhizotrons were filled with dark peat compost (Graberde, Plantaflor Humus Verkaufs-GmbH, Vechta, Germany) that had been processed in a wood chipper and then sieved in a drum sieve with 0.7 cm mesh size to make the compost more uniform and remove fibres. Half the rhizotrons were filled with this compost as processed, the other half was filled with compost that had been dried. This compost was dried by dispersing it thinly over a plastic cover in a glasshouse and leaving it to dry over approximately 1 week and then misted with water and mixed until at the appropriate moisture level. The moisture was measured using a KERN DBS Electronic Moisture Analyser (KERN & SOHN GmbH, Balingen, Germany) at 120°C until the moisture had been removed; the undried compost was measured at 63% gravimetric water content (control treatment), and the dried substrates were measured at 47% (reduced water treatment).

One-third of the rhizotrons with ‘control’ compost and one-third of the rhizotrons with dried compost then had compost removed to make a section at the top that was 15 cm wide (from the horizontal centre) and 12 cm deep (from the compost surface, 16cm from the top of the rhizotron). This section was then filled with the wheat precrop-treated soil (**Supplementary Figure 1**), collected from Bjertorp as detailed above. This was then repeated with another third of the rhizotrons but using the OSR precrop-treated soil. This resulted in 12 rhizotrons of each of the six soil-water treatment combinations: (1) OSR precrop-treated soil – Reduced Water, (2) OSR precrop-treated soil – Control, (3) Wheat precrop-treated soil – Reduced Water, (4) Wheat precrop-treated soil – Control, (5) Peat Compost – Reduced Water, and (6) Peat Compost – Control. The rhizotrons with the control water treatment were prepared and loaded into the GrowScreen-Rhizo 1 platform 3-8 days before the reduced water treatment (RWT). The GrowScreen-Rhizo 1 platform is divided into 4 blocks of 18 rhizotrons each, with blocks 1-2 on one side of the imaging cabinet, and blocks 3-4 on the other. Rhizotrons were loaded into each of the four sections of the platform in randomised blocks (**Supplementary Figure 2**), with each replicate set split equally on each side of the cabinet, in sequential order. The control rhizotrons were kept from drying out by watering with tap water every 24 hours with 100 ml of water spread over 1 minute. At transplantation, the drought-treated rhizotrons had 60 ml of water added to the area where the seedling was to be transplanted to reduce shock. However, due to the hydrophobic nature of the Peat Compost when dried, approximately 50 ml of compost was removed from the top in the centre (in a hemispherical shape) and replaced with ‘control’ compost and 60 ml of water. The control treatments had 1 litre of water applied on Day 0. An additional 60 ml of water was added to the drought treatments with the precrop-treated soil to account for differences in water levels at day 4.

100 seeds of winter wheat (*Triticum aestivum* L.), over 10 Petri dishes, were germinated for each of the two modern genotypes – ‘Brons’ and ‘Julius’, common cultivars in Swedish agriculture selected based on their differing RSA found in field conditions (Cope et al., 2024). Seeds were germinated between two pieces of filter paper per dish, with 2 ml of MiliQ water applied to each piece of paper. The dishes were then sealed with parafilm and then wrapped in groups with aluminium foil, before being kept in a growth chamber overnight with temperatures of 18/20°C night/day. Once germinated the most representative uniform seedlings were transferred into the rhizotrons by making a hole, approximately 2 cm deep, in the centre of the box surface and positioning the germinated seed in the hole with the coleoptile upward. The exposed top of the rhizotron was then covered with white plastic beads to reduce moisture escape and the control water treatment rhizotrons were watered every 12 hours with 200 ml of tap water over 2 minutes. The root systems were then photographed with the GrowScreen-Rhizo 1 (Nagel et al., 2012) facility three times a week starting on Day 3 after transplantation, using a high resolution camera (16 MP camera, IPX-16M3-VMFB, Imperx, Inc., Boca Raton, FL, USA; combined with Zeiss Distagon T 2,0/28 ZF-I lens, Jena, Germany) to acquire 230 mm per pixel images illuminated using LED-panels (LED Light Source SL3500-W-J, cool white, colour temperature 8000 K, Brno, Czech Republic).

We measured the shoot length (Days 7, 21, and 24), chlorophyll content (Days 10, 14, 17, 21, and 24) using a SPAD-502 (Konica Minolta, Tokyo, Japan), and chlorophyll fluorescence with estimates of phosphorus (P) and manganese (Mn) deficiencies (Days 17 and 21) using a P-Tester (SpectraCrop ApS, Hellevad, Denmark), alongside the growth stage at each measurement recording using the decimal growth stages (GS) (AHDB, 2021).

On Day 24, when roots reached the bottom of the rhizotron, the experiment was stopped and the material was collected from the rhizotrons. The shoot tissue was separated and the fresh weight was recorded, it was then placed in a paper bag. The root system was then separated from the soil and compost by washing with water. Roots were then soaked in water before washing again to remove the remaining substrate and they were placed in a paper bag. The shoot, root, and soil/compost were then dried for 14 days in a drying oven at 65°C before being weighed and recorded.

### 2.3 Nitrogen analysis

Due to low available plant biomass at harvest, the six replicates of each treatment combination (precrop soil and water treatment) were combined into three samples, paired by combining the highest and lowest weight, second highest and second lowest weight, and the two median weight replicates. These pooled samples were then milled to a particle size of 0.5 mm using a cyclone mill (RETSCH GmbH, Haan, Germany). The samples were then sent to an external company (Agri Lab AB, Uppsala, Sweden) for analysis, using a CN928 Series Macrop Determinator (LECO, St. Joseph, MI, USA) to assess the total nitrogen (N), using a modified method of the Soil quality - Determination of total nitrogen content by dry combustion [SS-ISO 13878; Swedish Institute for Standards (1996)].

### 2.4 Digital image analysis

Image analysis was undertaken using the GROWSCREEN-Root software (Nagel et al., 2009, 2012) along with a computer and pen display (Wacom Cintiq 22HD, Wacom Europe GmbH, Düsseldorf, Germany). Each image had the axial and lateral roots lined, in different colours, from which the coverage area was calculated; sequential images from the same rhizotron had the lines from previous measurements expanded upon. Data was measured in Pixels and converted to cm at a 55.53:1 ratio.

Additionally, this area was split into sections to assess root number distribution. A grid of 4383 × 3320 pixels (79 × 60 cm; H × W) was positioned over the sector of the image containing the soil/compost and split into 11 vertical and 14 horizontal sections (the last section containing the floral foam layer). The OSR and wheat precrop-treated soil additions occupied a section spanning the middle three vertical and top two horizontal sections.

### 2.5 Statistical analysis

Analysis for each of the root system architectural (**Supplementary Table 1**) and shoot traits measured, both non-destructive (**Supplementary Table 2**) and destructive (**Supplementary Table 3**), was done with a mixed-effects model, using the “proc mixed” function in the statistical software SAS (2018), for both the combined (where appropriate) and individual days. This was done with the data from all days for each variable a function of the fixed effects genotype, precrop treated-soil, water, and their interactions, while considering random effects for the replication nested within the block, and utilizing an unstructured covariance matrix for the repeated measurements over time (days). This was repeated for each day without the repeated element and without the combination with the time element in the model. Due to the heteroscedasticity of the models on the individual days (except for Leaf Area, Shoot fresh weight, Shoot dry weight, and Root dry weight) the data sets were transformed by using a log transformation and re-modelled. The fresh:dry shoot ratio, and the root:shoot ratio were both transformed by using a log transformation to remove distribution skew in ratios. A similar model was run on the axial and lateral root lengths using the data at day 24 divided into either the horizontal or vertical sections (**Supplementary Table 4**), with the genotype, precrop soil, and water treatment (plus their interaction) combined with the section position. Each model provided results from Type 3 Tests of Fixed Effect. Note: sample 202 (Julius, Wheat-Soil, Control) was removed as the plant was stunted from germination and died during the experiment, causing anomalous results. The nutrient data was also run using a modified version of the model, excluding the blocking factor due to the pooling of samples.

Visualisation of the analysed data was done using ‘R’ (R Core Team, 2013) packages ggplot2 (Wickham, 2016), ggpubr (Kassambara, 2023), and rmisc (Hope, 2013).

## 3 Results

### 3.1 Effect of water treatment

Water treatment had a significant effect on all destructive measurements (p<0.0001), with shoot traits including weight (fresh and dry) and leaf area limiting the plants in the reduced water treatment (RWT) to dry weights at 42% of the control, averaging over the three precrop soil treatments. The root dry weight was less affected by the RWT but still much reduced compared to the control, maintaining 57% of the root dried weight. The shoot-to-root ratio also differed between water treatments (p=0.0002), with the roots in the control treatment accounting for 24% of the biomass, vs. 33% in the RWT (**Supplementary Figure 3**).

The repeated root measurements were also significantly affected by the water treatment when taking time into account (p<0.0001; p = 0.0005 for root system depth). Most of the root metrics of the RWT were smaller compared to the control (except lateral root length). This was observed in the axial root length (with the relative RWT being 82% of the control in the beginning and 47% of the control towards the end), the total root length (82% to 51%), the root system depth (83% to 54%), and convex hull area (76% to 54%). The root system width was the least affected, though still significant, with the RWT maintaining 89-96% of the width of the control. Lateral root length showed the most variation, with the RWT having longer roots than the control in the earlier stages and the control treatment increasing to be larger by the end of the experiment (**Supplementary Figure 4**).

**Figure 1:**
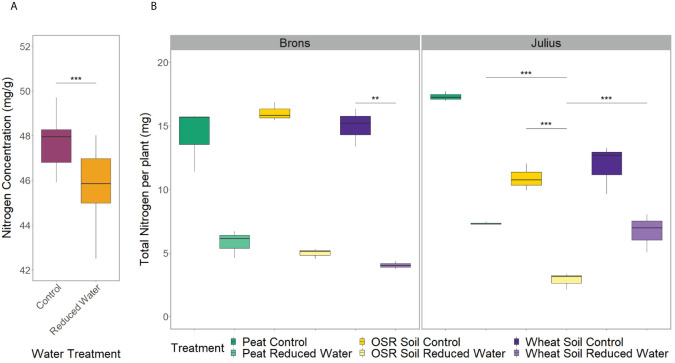
Spread of nitrogen data from the combined shoot and root material of two replicates. Comparing: **(A)** the water treatment, control (magenta) or reduced (RWT; orange), effect on the nitrogen concentration, and **(B)** the total treatment effect on the total plant nitrogen, separated by genotype, with the combined treatment including; precrop soil treatments of Peat compost (green), soil taken after OSR growth (yellow), or soil taken after wheat growth (purple); and the control (dark) or Reduced (RWT; light) water treatments. Significance is based on the Tukey pairwise comparisons adjusted p-value, with * = p>0.05, ** = p>0.01, and *** = p>0.001.

The repeated measurements of shoot characteristics showed differences in the shoot length, growth stage, and chlorophyll content (p<0.0001) between water treatments when factoring in time. This was seen in the later stages with the plants grown in the RWT having shorter shoot lengths and lower growth stages, but higher SPAD values (**Supplementary Figure 5**). The nutrient analysis showed differences in nitrogen concentration between water treatments (p=0.0005), with the control treatment having 4.2% more nitrogen concentration (**Figure 1A**). As the plants under RWT were smaller, the total plant nitrogen was also significantly different between water treatments (p<0.0001; **Figure 1B**).

### 3.2 Effect of precrop treated soil

**Figure 2:**
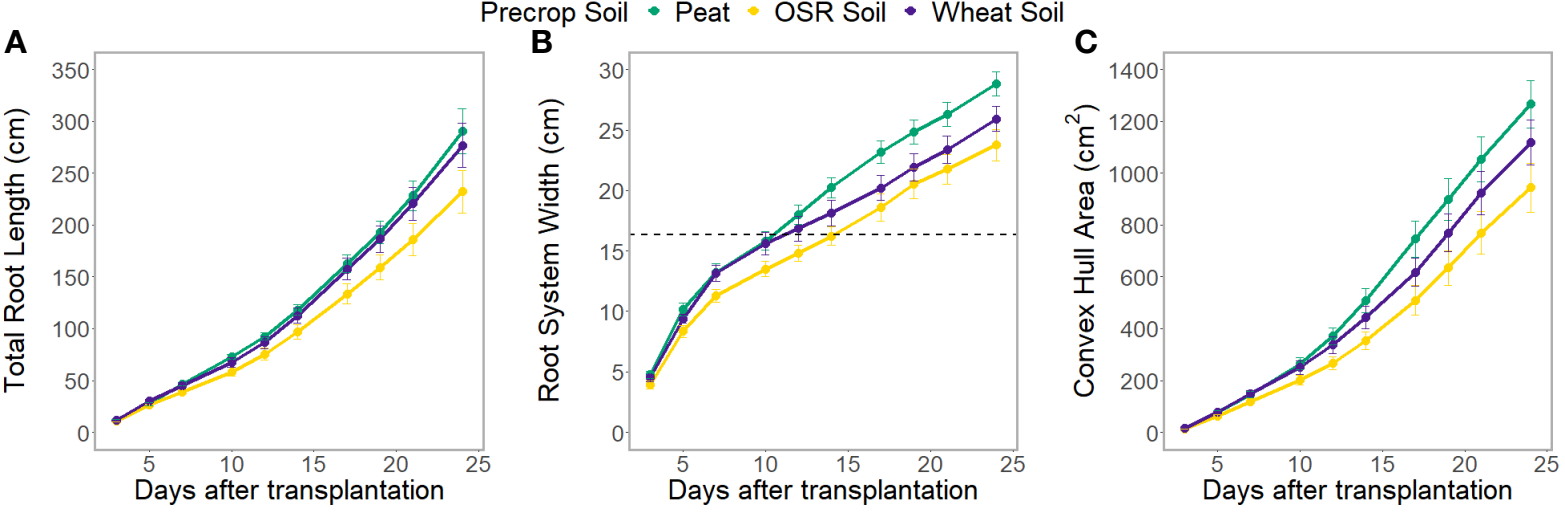
Average total root length **(A)**, root system width **(B)**, and convex hull area **(C)** at each time point, separated by the type of medium used as the precrop soil section; peat compost (green), soil taken after OSR growth (yellow), or soil taken after wheat growth (purple). The dashed line **(B)** indicates the width of the precrop soil addition where appropriate.

The root system architecture (RSA) traits measured all differed significantly between precrop soil treatments when taking time into account (p<0.0001). The peat compost treatment resulted in longer, and wider roots, and covered a larger area, than the OSR precrop-treated soil treatment (**Figure 2**), from days 7 (p=0.028), 5 (p=0.018), and 5 (p=0.031), respectively. The wheat precrop-treated soil treatment showed more similarities with the peat compost. Similar results were seen in the destructive measurement shoot traits taken on the final day, with the leaf area and shoot dry weight (**Supplementary Figures 6A, B**) both exhibiting higher averages in the peat compost compared to the soil. The shoot-to-root ratio was also affected by the precrop soil treatment (p = 0.014), with plants grown with a peat compost control developing a higher ratio of shoot biomass (**Supplementary Figure 6C**). Additionally, there were differences in the total plant nitrogen, taken on the final day, between precrop soil treatments (p=0.006) showing that plants with an OSR precrop-treated soil have lower total plant nitrogen than the peat compost, in a similar pattern to the shoot dry weight (**Figure 1**).

### 3.3 Combined precrop-water treatment effect

**Figure 3:**
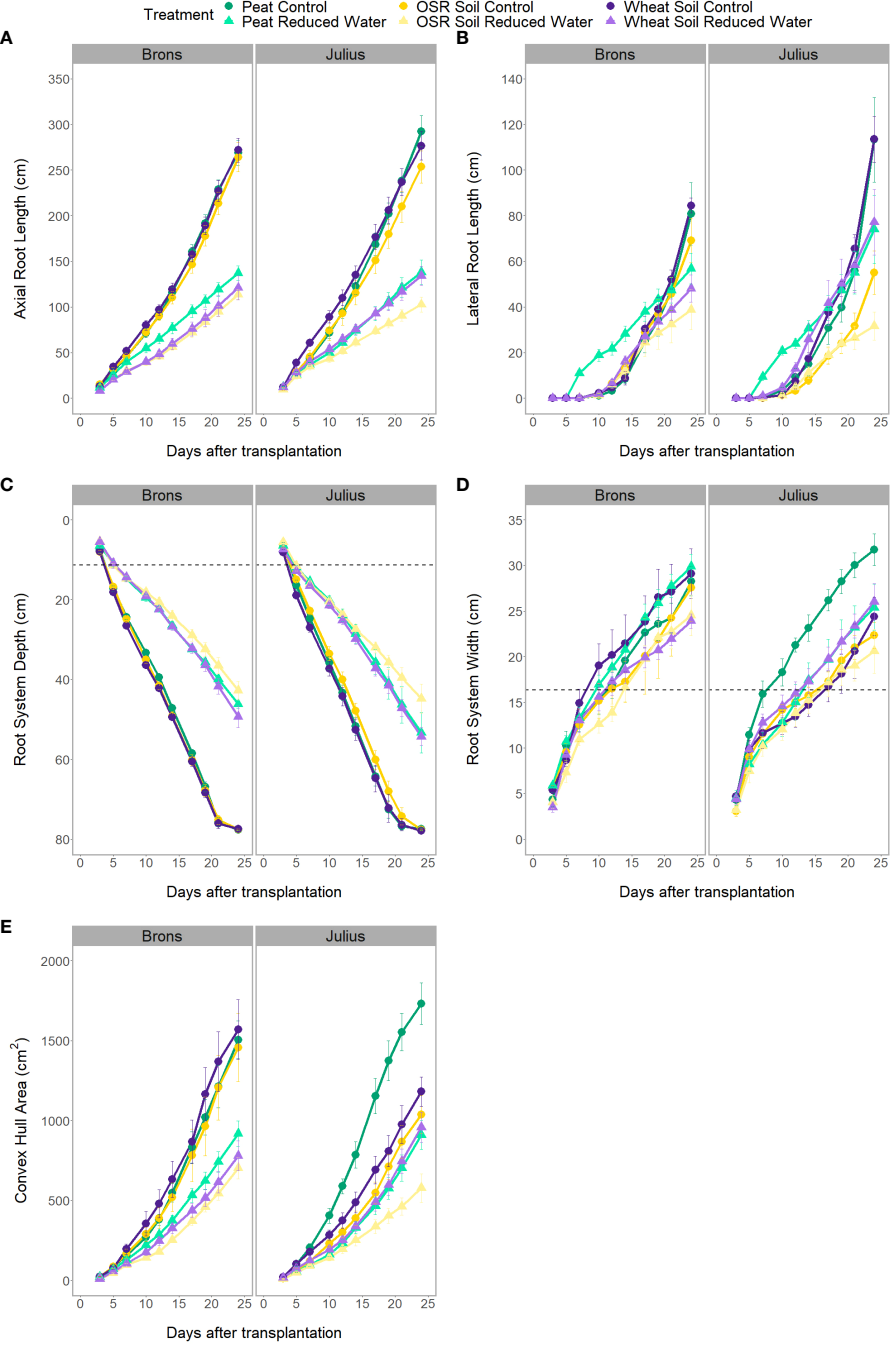
Progression of visible root traits over the different imaging dates of the rhizotron, for axial root length **(A)**, lateral root length **(B)**, root system depth **(C)**, root system width **(D)**, and convex hull area **(E)**. Data is separated by genotype (G – genotype) and divided by environment (E – precrop soil and water). Environmental divides are by colour for precrop soil – Peat compost (green), soil taken after OSR growth (yellow), or soil taken after wheat growth (purple) – with the dashed line **(B, C)** indicating the depth and width of the precrop soil addition. Shape and shade denoting water treatment – control as circles and darker shade, reduced (RWT) as triangles and in lighter colour shades.

There was a significant difference in the interaction of the precrop soil and water treatments (when coupled with time; **Figure 3**), for all RSA traits measured either independently, such as axial, lateral, and total root length (p<0.0001), or when factoring in genotype, such as root system depth (p = 0.0261), width, and convex hull area (p<0.0001). However, these differences were only seen between days 7-14 and were no longer seen on day 24, except for root depth. Like the effect of the precrop soil treatment on other RSA traits, root depth response to RWT showed similar differences between precrop soil treatments. Wheat precrop-treated soil and peat compost showed similar reductions in depth with the RWT (33-36%, respectively), whereas OSR showed a 44% reduction – based on similar control treatment depths. The interaction between the precrop soil and water treatment showed significant differences in the nitrogen concentration (p=0.027) that did not correspond to changes in dry weight. These showed differences between OSR and both peat and wheat precrop-treated soils, but only in with the RWT, not in control. Different from the dry weight which showed significant differences between the OSR precrop-treated soil and peat only in the control, and not between OSR and wheat precrop-treatments.

### 3.4 Genotype by treatment interactions

**Figure 4:**
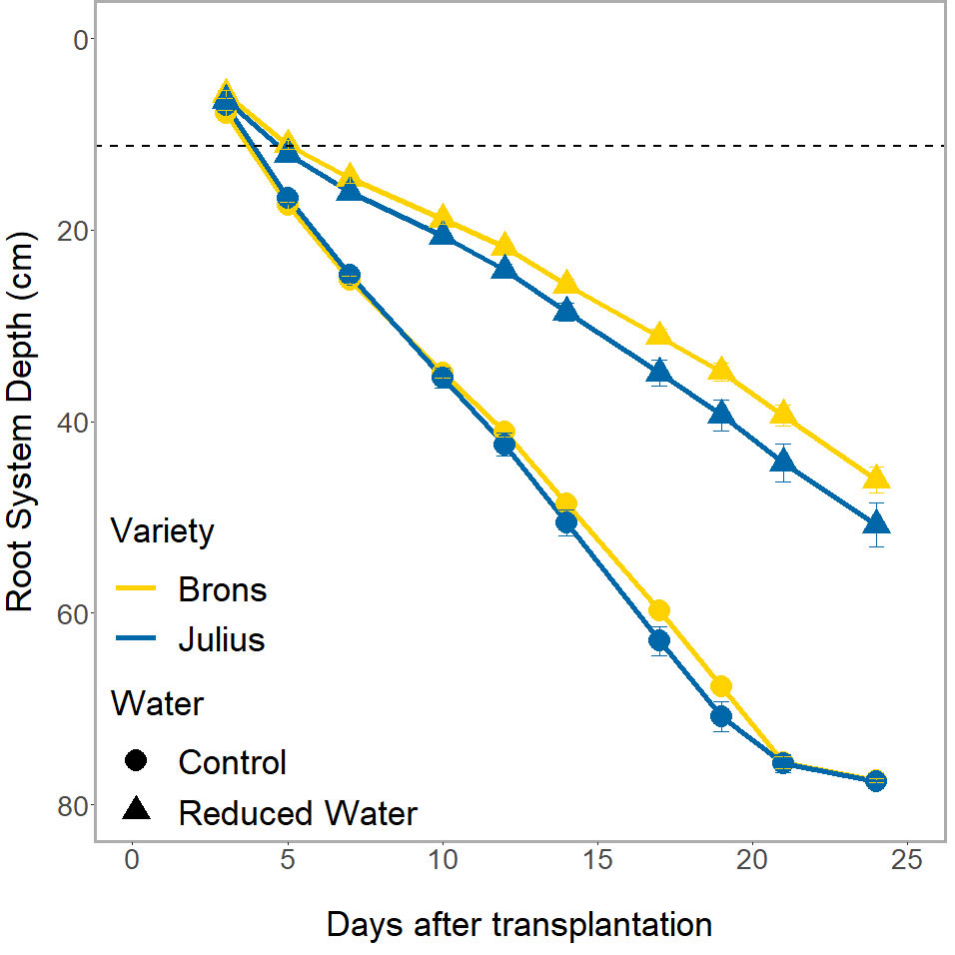
The total depth of the visible root system over the different imaging dates of the rhizotron. Divided by genotype (Brons in yellow, Julius in blue), and by water treatment (control as circles, reduced (RWT) as triangles). The dashed line indicates the depth of the precrop soil addition where appropriate.

The genotype effect on RSA, and the interaction with water treatment, was only seen in the depth of the root system (p ≤0.0023) when looked at in conjunction with the time factor (**Figure 4**). Whilst both genotypes had the same root system depths throughout the experiment during the RWT, Julius had a shallower depth at the control and thus was less affected by the RWT than Brons. This difference was seen throughout the experiment, with Julius having an average depth that is 8.8-13.1% deeper than Brons when under the RWT.

The interactions between the genotype and precrop soil factors were significant in all RSA traits measured (p<0.001) when taking time into account, except for lateral root length. The significant difference in the precrop soil-genotype interaction can be seen in **Figure 3A**, Brons showed no difference in axial root length between the different precrop soil treatments. In Julius, the peat compost and the wheat precrop-treated soil showed no effects, whereas plants with OSR precrop-treated soil had shorter axial roots than the peat compost in normal watering conditions and shorter than both the peat compost and the wheat precrop-treated soil in the RWT. Similarly, in the shoot traits, there was no significant difference in the leaf area or shoot dry weight in Brons, but Julius exhibited a smaller leaf area and less shoot dry weight with the OSR precrop-treated soil than the wheat precrop-treated soil and peat compost (**Supplementary Figure 6**). The total plant nitrogen also showed interactions between the genotype and precrop soil factors (p=0.0006) also limited to Julius, with the OSR precrop-treated soil having less total nitrogen than both other precrop soil treatments (**Figure 1B**).

#### 3.4.1 Combined treatment effect with genotype

Significant interactions between the genotype (G) and the environment (E – precrop soil and water) were shown, not only for the individual environmental treatments outlined above but for the interaction of both treatments with the genotype effect, when taking time into account in the model.

Significant differences between the G×E interaction were found in the root system depth (p = 0.0261). When looking at the root system depth, in controlled water environments, the root depths for both lines were the same, both between genotypes and between precrop soil treatments (**Figure 3C**). The differences were primarily in how the lines reacted to the precrop soil treatment when in the RWT. **Figure 3C** shows that there was a larger difference in Julius between the roots grown in the OSR precrop-treated soil and both the wheat precrop-treated soil and peat compost in the RWT, compared to Brons. Brons maintained 55% of the root depth found in the control water conditions on day 24 when grown in the RWT with OSR precrop-treated soil, with peat compost and wheat precrop-treated soil this was 59% and 64%, respectively. This significant difference was a result of Julius showing increased root depth preservation in RWT but in a precrop soil treatment-dependent manner, conserving 3% more in OSR precrop-treated soil, 6% more in wheat precrop-treated soil, and 9% more in peat compost.

Root system width had significant differences in the G×E interactions (p<0.0001) with each environment causing differential patterns in the genotypes. In most environments the genotype Julius had a narrower root system, however, this was not the case in the peat compost with the control water treatment, where it was wider, and the two soils with RWT where there was no significant difference (**Figure 3D**). These effects caused the patterns of the root system width adaptation to an environment to change with the genotype. In both genotypes, the root system width in the OSR precrop-treated soil followed similar patterns, with the RWT having slightly narrower roots, for Julius these roots were smaller and so the differences are not significant. The root system width after growing in the wheat precrop-treated soil showed a different trend depending on the genotype, with the control water treatments producing wider roots than the RWT in Brons but narrower roots than the RWT in Julius (approaching comparability towards the end of the experiment). The exact opposite was seen with the peat compost (**Figure 3D**).

The significant differences in the convex hull area, identified in the G×E interaction (p<0.0001), were due to a precrop soil treatment effect only being seen in Julius – except for a difference being seen between peat compost and OSR precrop-treated soil in the RWT (**Figure 3E**). Both lines exhibited a smaller convex hull area in the OSR during the RWT, however, it was much lower in Julius (60-63% of peat compost and wheat precrop-treated soil) compared to Brons (77-90%). With the control water treatment, in Brons, the two soil treatments produced a similar convex hull area to the peat compost treatment, whereas in Julius the two soil treatments had a smaller area, both in comparison to the peat compost and to Brons. A comparison of the changes caused by water limitation showed clear G×E interaction differences, namely from Julius’ reaction in wheat precrop-treated soil. In Brons, the convex hull area is reduced by 39-52% (Peat-OSR) when the RWT is applied, this is similar to Julius in peat compost (47%) and OSR precrop-treated soil (44%). However, the reduction in convex hull area for Julius in the RWT with a wheat precrop-treatment was only 19%, compared to the 50% in Brons.

We also found significant differences in the total plant nitrogen (p= 0.004) when factoring in the G×E interactions. Similar to the soil precrop and water treatment interaction, this did not correspond to the shoot dry weight. In both lines, there was one precrop soil treatment which showed significant decreases in total nitrogen when under a RWT, in Brons this was with wheat precrop-treated soil, in Julius it was with OSR precrop-treated soil (**Figure 1B**).

**Figure 5:**
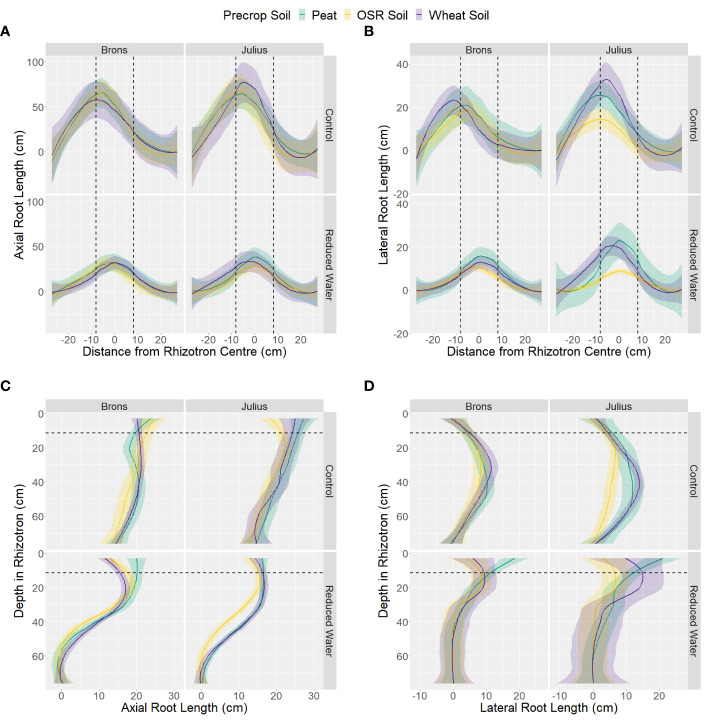
Distribution of length of axial **(A, C)** and lateral **(B, D)** roots, on the last measurement day (24), within the different areas of the box, split into either 11 sections horizontally **(A, B)** or 14 sections vertically **(C, D)**, with the average distance from centre or depth (respectively) taken for each box. Data is divided by the type of medium used as the precrop soil section; Peat compost (green), soil taken after OSR growth (yellow), or soil taken after wheat growth (purple). The dashed black lines represent the distance from the centre, or depth (respectively) that the precrop soil section ended.

Analysis of the data by section showed little difference in the horizontal distribution between the G×E combinations for both axial and lateral root lengths (**Figures 5A, B**). Differences in the horizontal position only significantly interacted with water treatment (p-values <0.0001), by having higher proportions in the centre section when under RWT, and with the genotype-precrop soil interaction (p=0.041) in the lateral root length. Larger differences were seen in the vertical distribution of both axial and lateral roots (**Figures 5C, D**). Differences in vertical position also significantly interacted with water treatment (p-values <0.0001). The control treatment had more axial root length in each section, but this difference was smaller closer to the top. This was similar in the lateral root length, however, the RWT had more lateral roots in the top and precrop soil section, as expected. Differences from the precrop-treatment between sections were also seen (p-values <0.0001), with OSR having lower axial and lateral roots in only the middle section – except lateral roots in the precrop soil which were different between all treatments.

## 4 Discussion

This study is the first assessment of the precrop effect using a rhizotron facility, simulated through precrop-treated soil, on the early growth of the root system and its consequences for the RSA as a whole, both with and without water limitation. Clear effects on the RSA from this small amount of precrop-treated soil around the seedling show the success of this method in generating a precrop effect, in part, in laboratory conditions. From this study, we can see that the water stress shows expected effects on the winter wheat, with the reduced water treatment (RWT) causing reduced plant size, as outlined by the reduced biomass, length, and growth stage, as expected (Bektas et al., 2023). The RWT did show a larger effect on the shoot biomass, more than the root biomass, indicating that the root system was less reduced in size than the shoots, suggesting increased prioritisation of the root system during water stress; this is supported by the significant difference in the shoot-to-root ratio. These results support previous findings showing increased percentages of root biomass when under drought conditions (Bektas et al., 2023), however, this could be linked to the lower growth stage of the RWT, which affects this ratio (Fageria, 1992). The RWT also showed decreased concentrations of nitrogen per biomass, which could indicate reduced uptake associated with drought conditions. The SPAD measurements seemed to indicate increased leaf N content per leaf area in drought conditions. However, SPAD measurements are sensitive to variation in specific leaf area (Peng et al., 1993), and increased leaf N per area in response to drought has been reported previously (Xiong et al., 2015) and has also been related to drought adaptation (Weih et al., 2011). The RSA measurements showed that the RWT caused a reduction in the root depth and area, as well as the total root length. Whilst reduced area and total root length were expected, previous studies have shown drought stress to cause deeper rooting (Barraclough et al., 1989). This could be due to the Barraclough et al. (1989) experiment running for a longer period, or due to the mechanical constraints of the rhizotron boxes preventing deeper rooting being triggered, but could also indicate that these genotypes are drought sensitive as drought tolerant lines tend to respond to low water stress by promoting deeper rooting (Karlova et al., 2021). Additionally, the control treatment showed more axial roots at each section depth, with only the lateral roots in the top sections showing an increased number with RWT. This similarly shows that the lines chosen are drought sensitive as drought-tolerant lines tend to have reduced lateral root density, as well as low rooting density in shallow soils and high rooting density in deeper soils (Zhan et al., 2015).

### 4.1 Precrop-treatment effects on early RSA and the effect of RWT (hypotheses H1 & H2)

Shoot differences were seen to be affected largely by the substrate type, with higher leaf area and shoot dry weight, therefore affecting the shoot-to-root ratio, in the peat compost vs. the two soil treatments. This is likely due to higher levels of nutrients, such as nitrogen (N) and phosphorus (P), probably in the peat compost compared to the soil samples (Kumar et al., 2020). However, differences between the preceding crops of the two soils were seen when looking at the RSA. Whilst most of the studies between different precrops are focused on biopores (Han et al., 2016), other studies have similarly shown differences in the effect of precrop on the RSA (Sieling et al., 2005; Seidel et al., 2019). The soil with a wheat precrop-treatment showed a similar RSA to the peat compost control, whereas the soil with the OSR precrop-treatment showed narrower root systems with less total root length, and thus less convex hull area, and fewer roots at the middle depth. This is in contrast to other studies which have shown that smaller root systems after a wheat precrop compared to an OSR precrop (Sieling et al., 2005), possibly due to microbial or chemical changes (Angus et al., 2015). However, Ryan et al. (2003) have shown OSR underperforming as a preceding crop, particularly when under dry conditions, with a correlating reduction in wheat root growth. This supports our hypothesis (H1) that the preceding crop-treated soil will affect the early stages of root development, resulting in differences in RSA, though it was expected that this difference would be reversed from what is presented. This experiment used different precrop-treated soils only in the immediate area of developing seedling, using a common medium between treatments for the rest of the root growth, showing that the conditions of the soil at the early stages of root growth are important for the structure of the whole root system. The hypothesis that precrop-treated soil will affect the changes in RSA caused by water stress (H2) is also supported, to a limited extent. We can see similar patterns between the precrop-treatments for the response of root system depth to RWT, with OSR precrop-treatment resulting in a more affected root system. This supports the data from Ryan et al. (2003) that shows OSR as a precrop can be detrimental in dry conditions. It also supports other findings that show yield penalties of certain precrops during drought (Nie et al., 2022) or with different levels of benefit (Seidel et al., 2019). Other RSA traits are only affected by the RWT differently depending on precrop-treatment in the second week of the experiment and are not seen on the final day. This is possibly due to the growth of the roots outside of the precrop-treated soil, these differences could have been prevalent in the later growth stages with continuous contact with the precrop-treated soils.

### 4.2 Genotypes react differently to the soils from different precrops (hypothesis H3)

Differences were seen between the two genotypes, as expected from previous studies on these genotypes (Cope et al., 2024), and others (Nguyen and Stangoulis, 2019). However, differences were also seen in interactions between precrop-treatment and genotype, with the genotype Brons showing similar variables between precrop soil treatments, but Julius showing differences between the precrop-treatment seen overall, with the OSR precrop-treated soil resulting in shorter roots, smaller leaf area, and less shoot dry weight. This supports results from other studies that show genotype-specific yield differences after different precrops (Fekete and Pepó, 2019). The difference in the G×E interaction is intensified when taking into account the water treatment. This is shown in the root width, with Brons showing wider roots in the control (compared to the RWT) only after wheat precrop-treated soil, whereas Julius shows wider roots in the control only after peat. This narrowing of the RSA when under RWT was expected, as narrower and deeper root systems tend to develop during drought stress to access deeper water reserves (Zhan et al., 2015; Karlova et al., 2021), as well as other environmental pressures (Robinson et al., 2018). However, this occurring only in certain soils that differ depending on the genotype was not expected, indicating an interaction between soil and precrop type that prime the roots for differences, which could affect the suitability of the crop to environmental stresses. Similarly, differences in the total plant nitrogen show that Brons was affected by the RWT after a wheat precrop-treatment, and Julius after OSR. The differences in the effect of the RWT on the convex hull area between precrop soil treatments are also genotype-specific, seen only in Julius. Reductions in the convex hull area caused by the RWT are similar in Brons, but, in Julius, the reduction after wheat precrop-treated soil is much less compared to both the other precrop soil treatments and Brons. The reason for this difference could be due to several factors, such as the different genotypes favouring microbes that are present in the soil after one precrop more than the other, nutrient differences caused by the preceding crop that one genotype has adapted to but the other has not, soil structures from one precrop which are utilised by one genotype and not the other (Smith and De Smet, 2012). This indicates that certain genotypes might benefit from different precrops in ways that other genotypes might not. These differences in the genotypic-specific response to the environmental effects of precrop-treatment and water stress support our final hypothesis (H3) and show that crops can be genetically adapted to produce root traits that will favour different environmental pressures (Karlova et al., 2021; Gao et al., 2023), both natural and designed.

### 4.3 Implications for future farming and breeding

The data from this study suggest that the precrop and the weather conditions play an important role in determining which genotypes will produce a better root system, allowing for greater potential yield (Richards, 2008) and yield resilience (Ober et al., 2021). This can be seen clearly in the data presented here where Julius showed less root area decline with RWT after a wheat precrop-treatment compared to other precrop soils, whereas this was not seen in Brons. Further understanding of the mechanisms that cause these genotype-specific differences is needed to better predict the effect of soil and farm management practices on RSA, and how this is affected by climate change-associated stresses such as drought. This understanding will allow for the suggesting and breeding of crop genotypes that are better suited to specific farm systems, such as rotational cropping, that can cope well with stresses such as drought. Additionally, whilst an OSR precrop-treatment may increase yield in a field setting, the early root growth seen with an OSR precrop-treatment in this experiment suggests that wheat after OSR may be more sensitive to stresses due to the reduced root growth. This is seen in both genotypes but is more prevalent with Julius, and shows greater effects of the RWT. Further testing on this effect with multiple genotypes to assess the extent the elite genotype population is affected is needed, and an assessment to test how much this affects yield resilience under drought conditions is needed, complementing the work from Ryan et al. (2003). The difference in precrop treatment found in these results shows that the use of precrop-treated soil to generate the precrop effect, in part, in a laboratory setting is a valid and promising method. This method can be used for more in-depth screening of genotypes for sensitivities to different precrop effects, in different simulated environments.

Identification of markers in genotypes or other lines which produce RSAs favourable for different environmental combinations will allow for marker-assisted breeding of crops with RSA that give better and more resilient yields under new management practices and in the face of changing climate (Fradgley et al., 2020). By introducing a diverse range of genotype and management practice-specific crop resilience traits into the elite genotype’s population we could help increase crop stability in the face of climate change and rectify yield stagnation seen in Europe (Kahiluoto et al., 2019). One complication in this breeding that could arise is that vernalisation genes have been shown to affect RSA, increasing the challenges when breeding for specific RSA traits in winter wheat (Voss-Fels et al., 2018). Additionally, information on how the genotype-specific RSA is affected by other interactions, such as other abiotic stresses, including nutrient availability, and biotic stresses, as well as benefits from the microbial communities, is needed. This will give a greater understanding of the effect the environment will have on genotypes, and in selecting which would be best for different environments. Increasing the number of soil compositions this is tested in is also necessary to adapt a genotype’s RSA to a given region, as different soils will affect the RSA and its interactions differently (Rich and Watt, 2013).

Our findings show that a crop’s RSA reaction to drought stress is a result of a combination of genetics, environment, and management practices. This has an impact on the study of the precrop effect on crop performance and yield and could explain the negative, or non-positive, effect of precrop in drought conditions (Ryan et al., 2003; Nie et al., 2022). By understanding how the gene expression influencing RSA is affected by the different mechanisms of the precrop effect, and how that is changed in drought conditions, we will form an understanding of the mechanistic approaches that trigger genotype-specific RSA that will benefit the crop. Employing the techniques showcased in this experiment will streamline the screening of numerous lines, enabling a thorough evaluation of the impact of diversity in cropping systems on early RSA development. This, in turn, will facilitate the identification of genomic factors corresponding to favourable reactions. The identification of markers associated with these mechanisms will allow breeding programs to incorporate specific root characteristics, tailoring the genotypes to maximise yield stability under combinations of management and stress.

### 4.4 Conclusions

Our results show that the preceding crops affect early root growth differently, changing the RSA, and depending on the water stress (supporting H1&2). Furthermore, there is a difference in this reaction between the two genotypes (supporting H3) that had been chosen for their different RSA. Additionally, we have shown a viable method for generating a precrop effect using only a small section of precrop-treated soil around the seedling in laboratory conditions. This method will enable a higher throughput assessment of lines and conditions that will broaden our understanding of root development changes due to crop diversification.

Studies to uncover the mechanisms of this interaction could allow for better selection practices for rotational cropping, as well as breeding lines specifically suited for these environments and that are more climate-proof. This can be done using the precrop-treated soil method, outlined here, to screen crops and environments, and then marker-assisted selection to identify associated genetic regions. Environment-tailored genotypes can increase yield and yield stability on the farm level, but also increase the climate change readiness of the agricultural system by providing genotype diversity, which acts as a buffer against unpredictable environments created by climate change.

## Data availability statement

The raw data supporting the conclusions of this article will be made available by the authors, without undue reservation.

## Author contributions

JC: Conceptualization, Data curation, Formal analysis, Investigation, Methodology, Visualization, Writing – original draft, Writing – review & editing. FB: Investigation, Validation, Visualization, Writing – review & editing. AG: Methodology, Resources, Software, Supervision, Writing – review & editing. JL: Methodology, Resources, Writing – review & editing. KN: Conceptualization, Resources, Supervision, Writing – review & editing. FF: Funding acquisition, Resources, Supervision, Writing – review & editing. MW: Conceptualization, Methodology, Supervision, Validation, Visualization, Writing – review & editing.

## Supplementary material

Supplementary Figure 1 Image of one rhizotron with the precrop-treated soil, outlining the dimensions of the rhizotron and the soil treatment.

Supplementary Figure 2 Representation of the position of the replicated treatment rhizotrons and genotypes. The 72 rhizotrons were split into four blocks of 18. The imaging box and track were separating blocks 1-2 from 3-4. Between the two blocks on each side, there was an additional gap.

Supplementary Figure 3 Comparison of the effect of the water treatments, control (magenta) and low (RWT; orange), on the destructive shoot and root measurements at the end of the experiment. Measurements include leaf area **(A)**, shoot weight (fresh – **B**, and dried – **C**), and root weight (dried – **D**).

Supplementary Figure 4 Comparison of the effect of the water treatments, control (magenta) and low (RWT; orange), on repeated root measurements during the experiment. Measurements include Root lengths (Axial – **A**, Lateral – **B**, and Total – **C**), Root system depth **(D)** and width **(E)**, and the Convex hull area **(F)**. These are faceted by the day in the experiment these measurements were taken. The dashed lines **(D)** and **(E)** indicate the depth and width (respectively) of the precrop soil addition.

Supplementary Figure 5 Comparison of the effect of the water treatments, control (Magenta) and low (RWT; Orange), on shoot measurements during the experiment. Measurements include **(A)** shoot length – from the base of the plant to the tip of the shoot, **(B)** the growth stage - as outlined in AHDB (2021), and **(C)** the relative chlorophyll content – displayed as SPAD value. These are faceted by the day in the experiment these measurements were taken.

Supplementary Figure 6 Spread of final day shoot characteristics – leaf area **(A)**, shoot dry weight **(B)**, and shoot-to-root ratio (**C**; Log) – separated by genotype and divided by the type of medium used as the precrop soil section; Peat compost (Green), soil taken after OSR growth (Yellow), or soil taken after Wheat growth (Purple).

Supplementary Table 1 P-value data of the repeated root measurements from a mixed-effect model with variables genotype, soil, and water treatments (plus their interaction) for each day, plus the combined analysis with all variables combined with the time element (day). The P-values highlighted (green) are those that are classified as significant (P>0.05).

Supplementary Table 2 P-value data of the repeated shoot measurements from a mixed-effect model with variables genotype, soil, and water treatments (plus their interaction) for each day, plus the combined analysis with all variables combined with the time element (day) for those with more than two time-points. The P-values highlighted (green) are those that are classified as significant (P>0.05).

Supplementary Table 3 P-value data of the destructive shoot and root measurements from a mixed-effect model with variables genotype, soil, and water treatments (plus their interaction) for the last (24^th^) day of the experiment. The P-values highlighted (green) are those that are classified as significant (P>0.05).

Supplementary Table 4 P-value data of the root length measurements from a mixed-effect model with variables genotype, soil, and water treatments (plus their interaction) for the last (24^th^) day of the experiment, divided into either three horizontal **(A)** or four vertical **(B)** sections. The Centre and Soil sections of the Horizontal and Vertical sectioning, respectively, contain the treated soil sections. The P-values highlighted (green) are those that are classified as significant (P>0.05).
