# Supplementary figures and images for: Precrop-treated soil influences wheat (Triticum aestivum L.) root system architecture and its response to drought
Source: Front Plant Sci. 2024 Jun 4;15:1389593. doi: 10.3389/fpls.2024.1389593 (PMC11184070; doi:10.3389/fpls.2024.1389593)

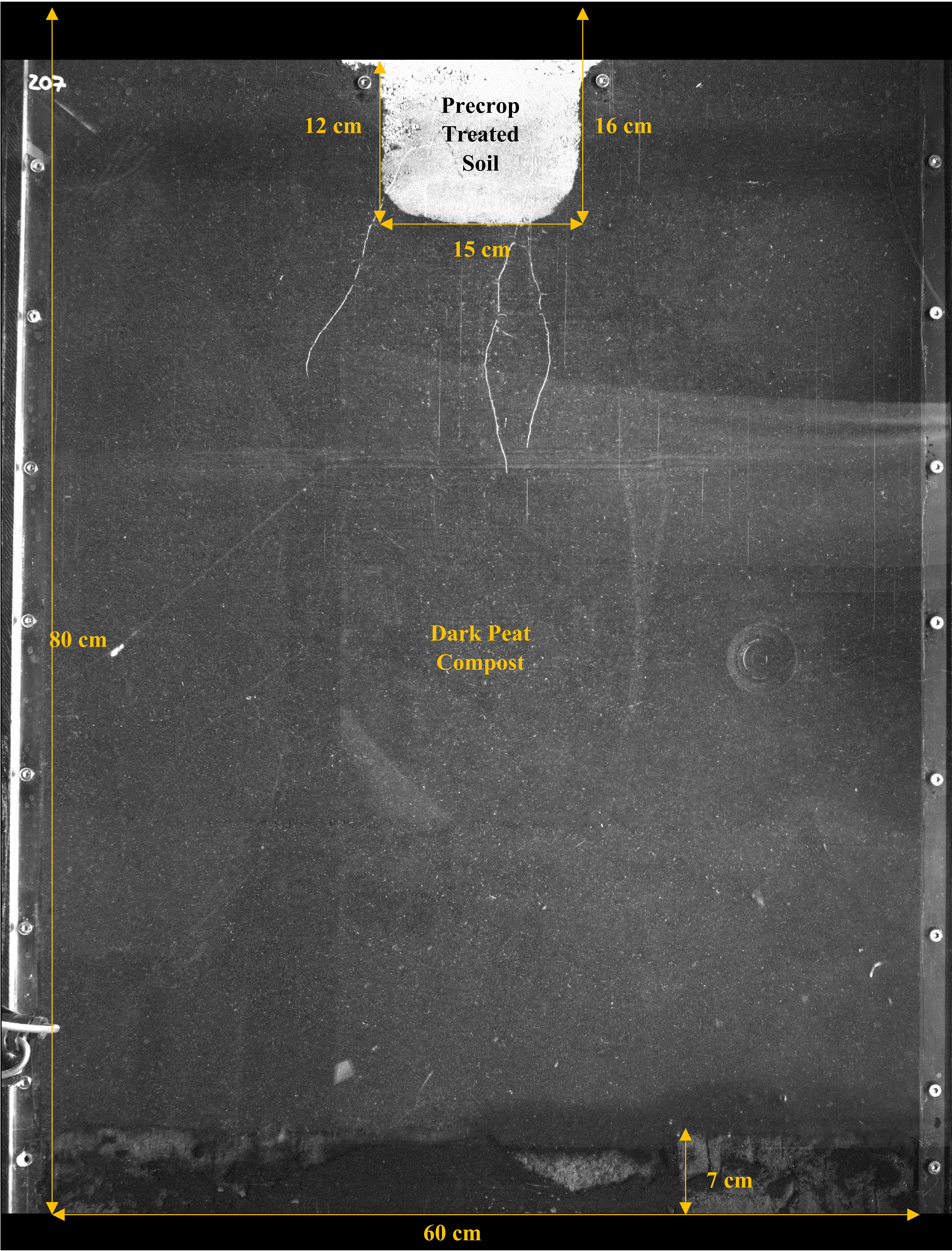

Supplement: Supplementary Figure 1 — Image of one rhizotron with the precrop-treated soil, outlining the dimensions of the rhizotron and the soil treatment. [file Image_1.jpeg]

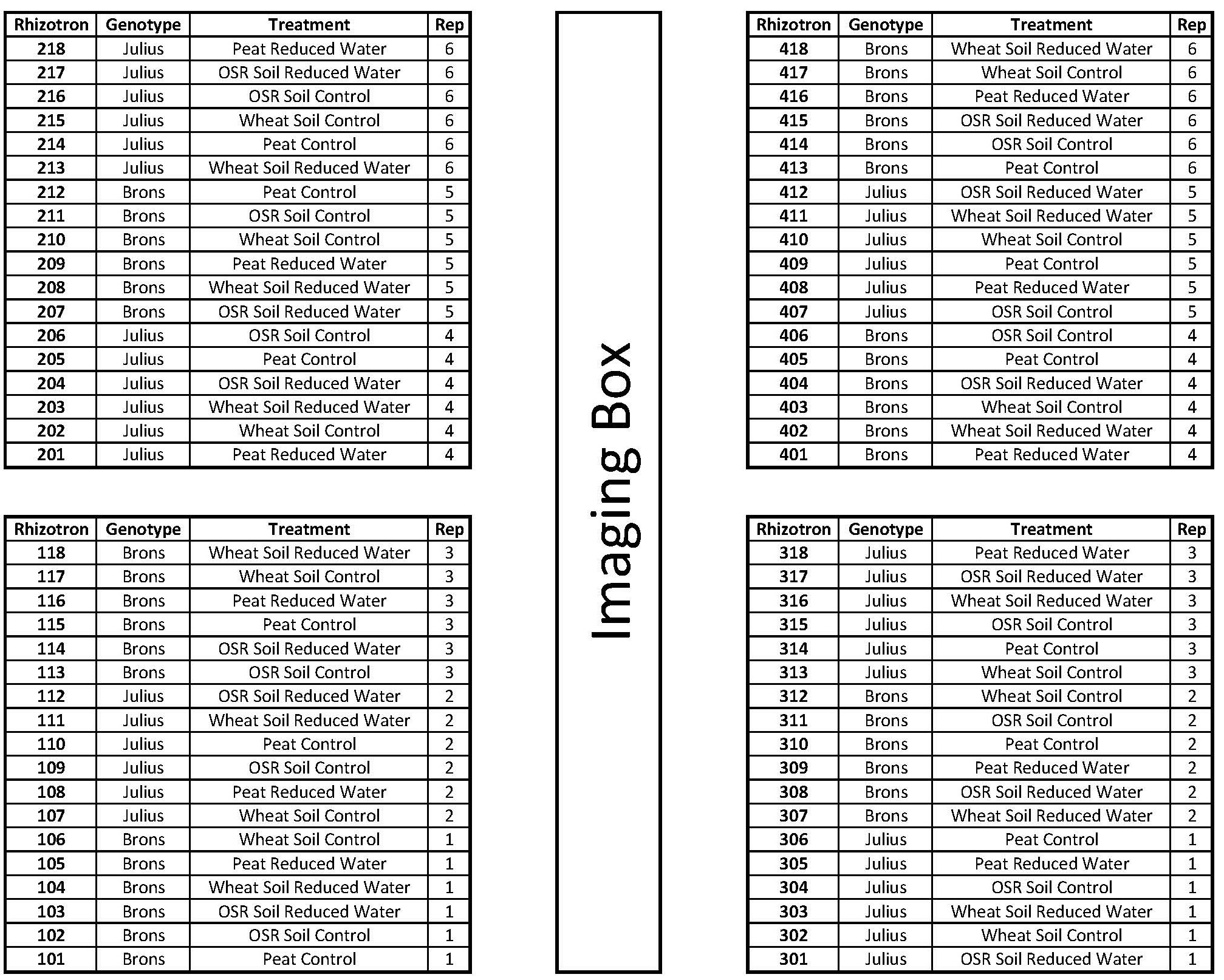

Supplement: Supplementary Figure 2 — Representation of the position of the replicated treatment rhizotrons and genotypes. The 72 rhizotrons were split into four blocks of 18. The imaging box and track were separating blocks 1-2 from 3-4. Between the two blocks on each side, there was an additional gap. [file Image_2.jpeg]

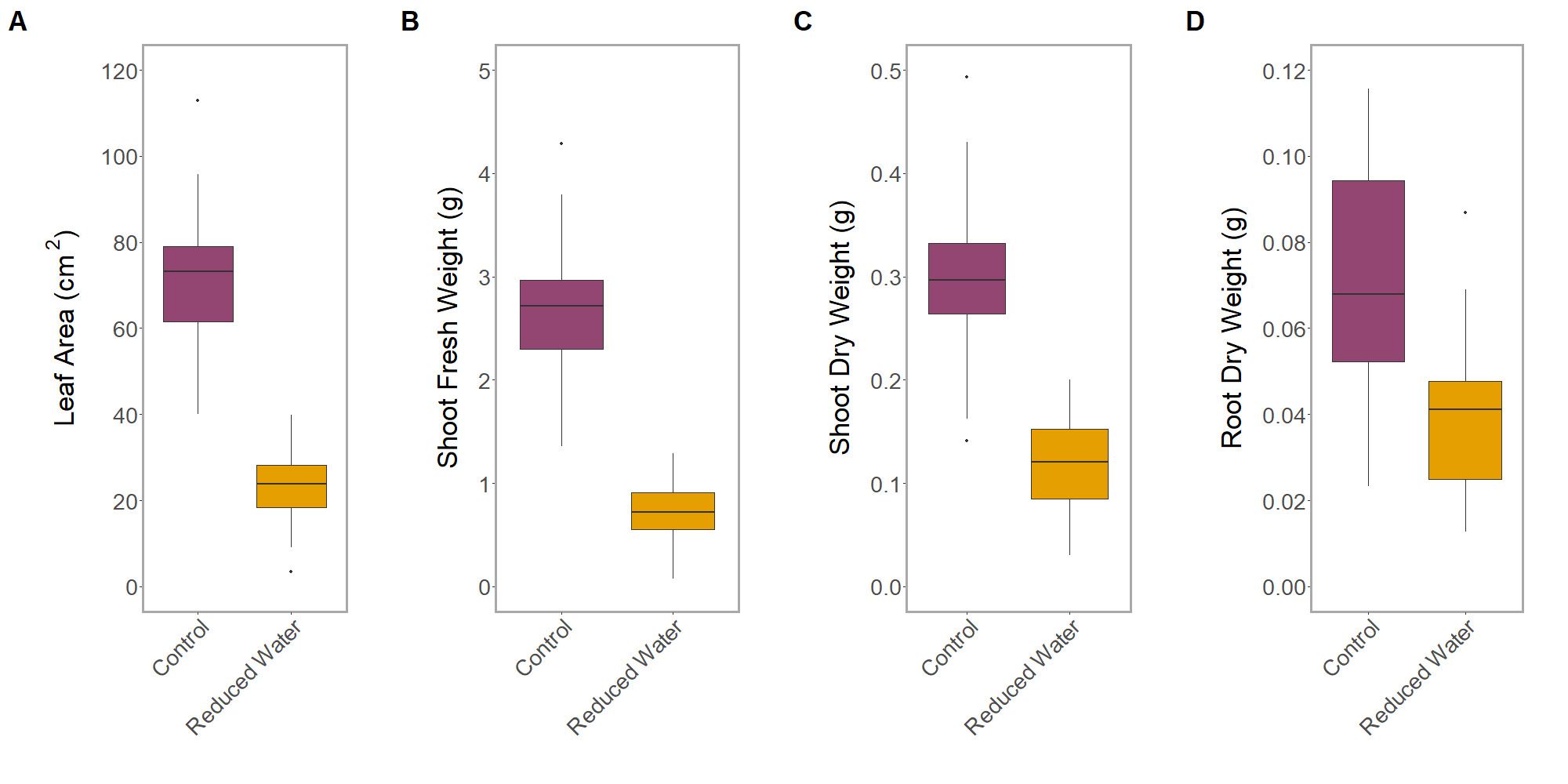

Supplement: Supplementary Figure 3 — Comparison of the effect of the water treatments, control (magenta) and low (RWT; orange), on the destructive shoot and root measurements at the end of the experiment. Measurements include leaf area (A), shoot weight (fresh – B, and dried – C), and root weight (dried – D). [file Image_3.jpeg]

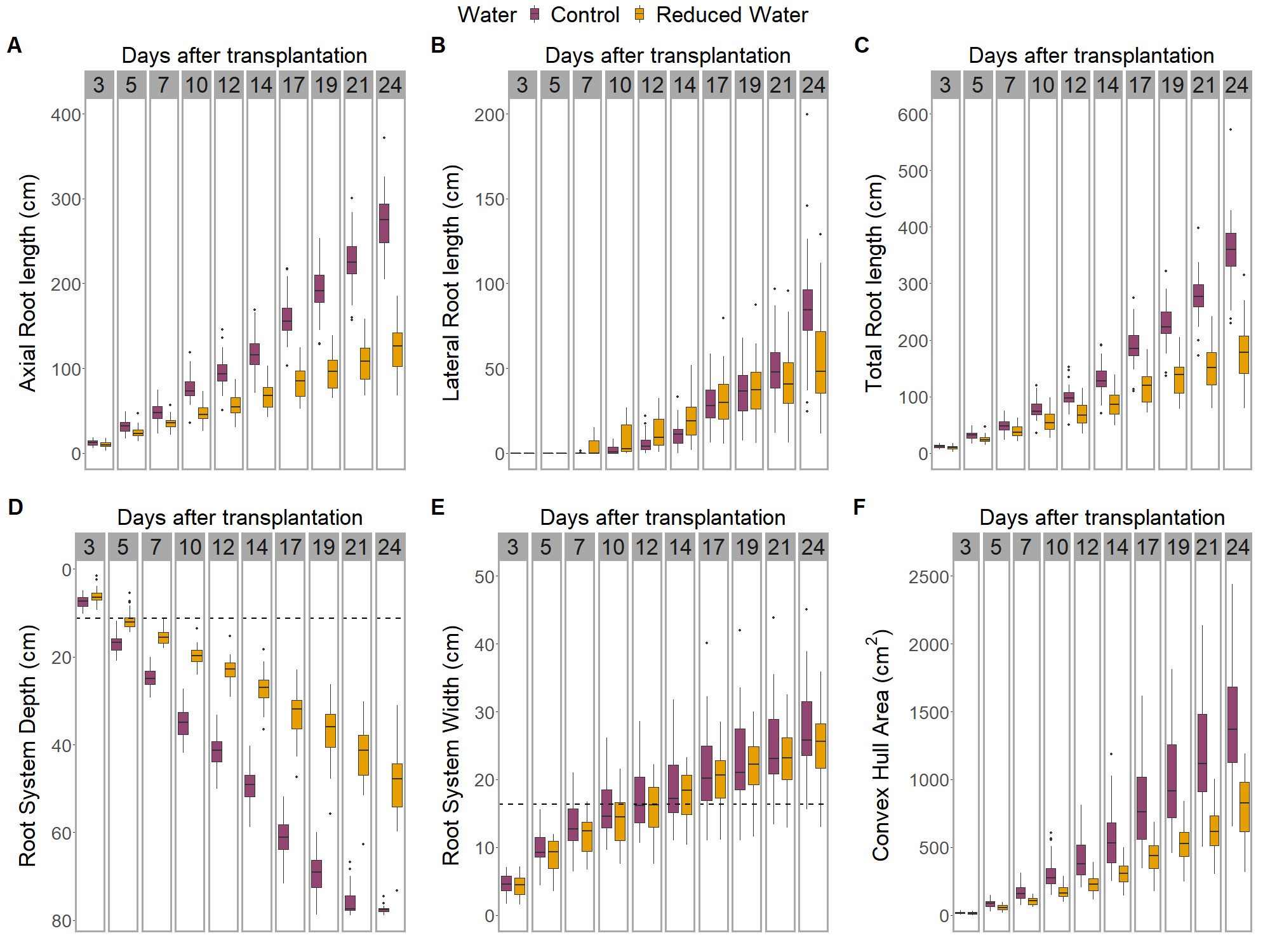

Supplement: Supplementary Figure 4 — Comparison of the effect of the water treatments, control (magenta) and low (RWT; orange), on repeated root measurements during the experiment. Measurements include Root lengths (Axial – A, Lateral – B, and Total – C), Root system depth (D) and width (E), and the Convex hull area (F). These are faceted by the day in the experiment these measurements were taken. The dashed lines (D) and (E) indicate the depth and width (respectively) of the precrop soil addition. [file Image_4.jpeg]

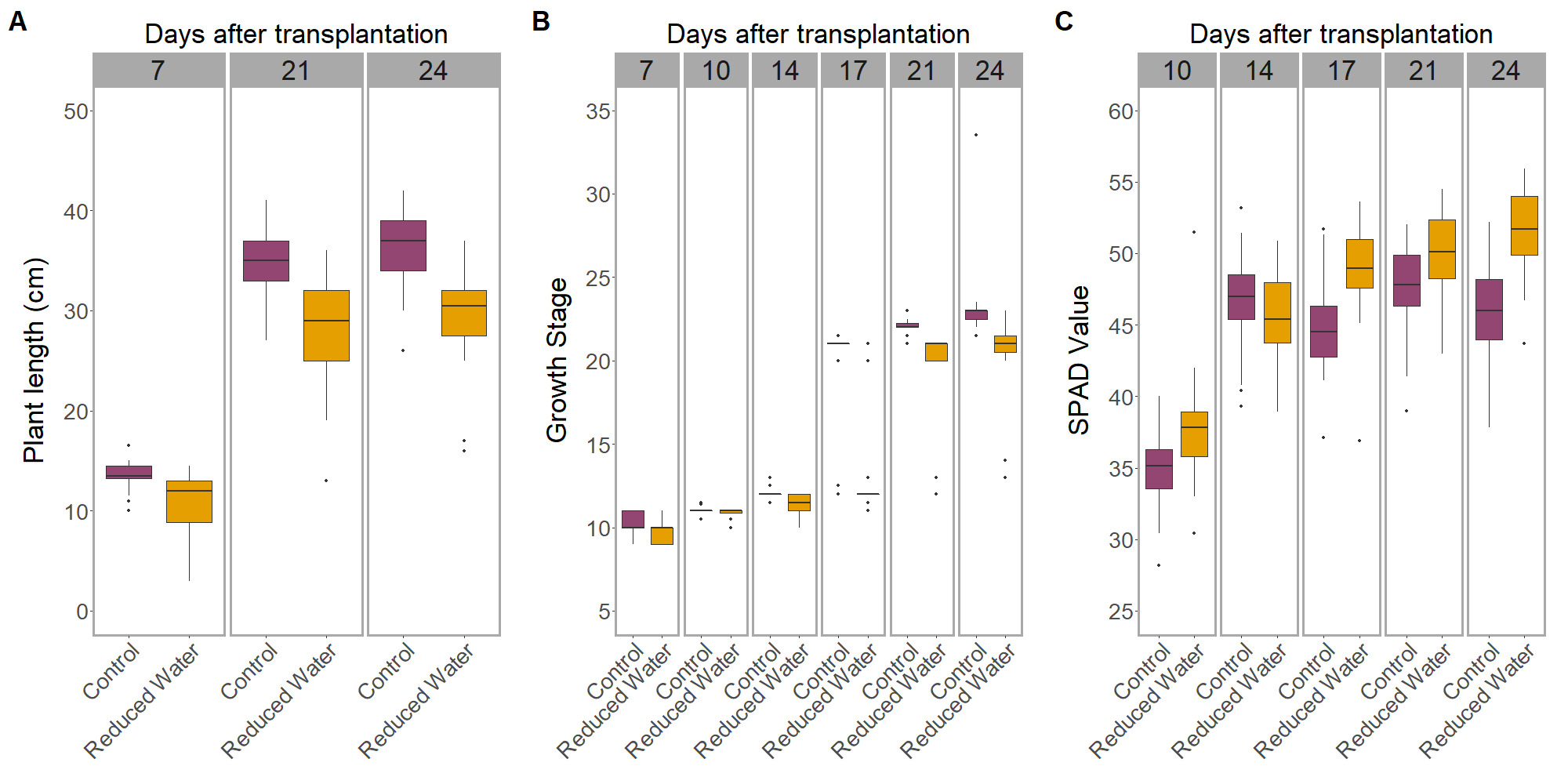

Supplement: Supplementary Figure 5 — Comparison of the effect of the water treatments, control (Magenta) and low (RWT; Orange), on shoot measurements during the experiment. Measurements include (A) shoot length – from the base of the plant to the tip of the shoot, (B) the growth stage - as outlined in AHDB (2021), and (C) the relative chlorophyll content – displayed as SPAD value. These are faceted by the day in the experiment these measurements were taken. [file Image_5.jpeg]

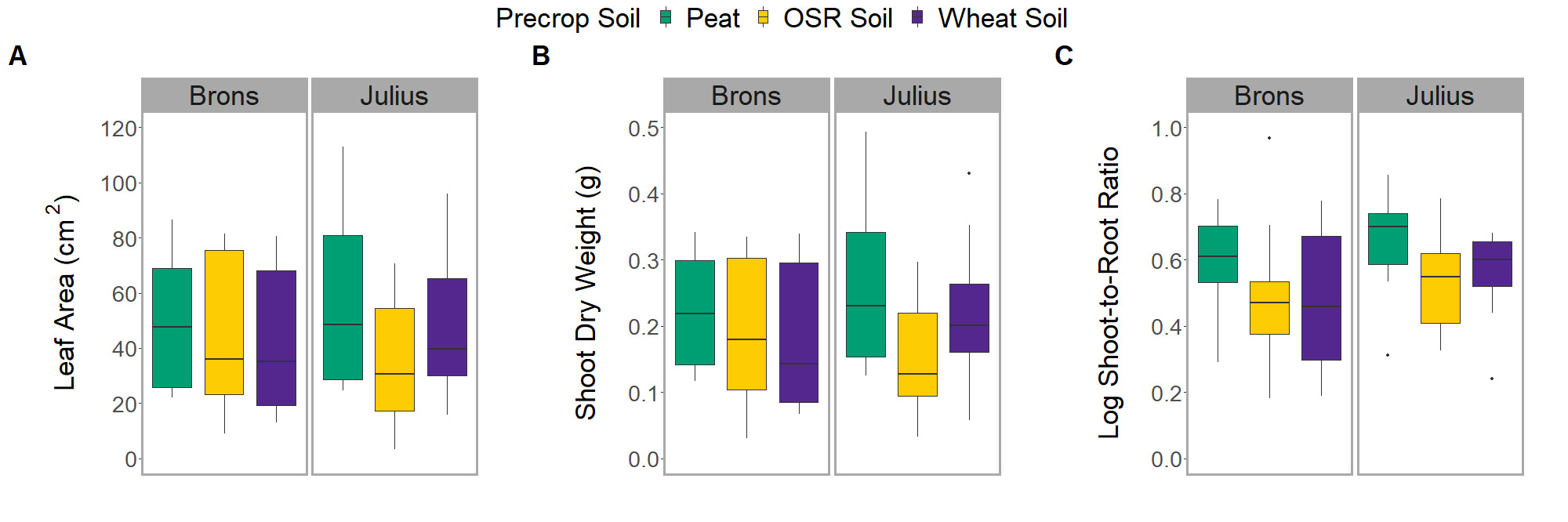

Supplement: Supplementary Figure 6 — Spread of final day shoot characteristics – leaf area (A), shoot dry weight (B), and shoot-to-root ratio (C; Log) – separated by genotype and divided by the type of medium used as the precrop soil section; Peat compost (Green), soil taken after OSR growth (Yellow), or soil taken after Wheat growth (Purple). [file Image_6.jpeg]
