# Supplementary material for: Precrop-treated soil influences wheat (Triticum aestivum L.) root system architecture and its response to drought
Source: Front Plant Sci. 2024 Jun 4;15:1389593. doi: 10.3389/fpls.2024.1389593 (PMC11184070; doi:10.3389/fpls.2024.1389593)
Supplement: Supplementary Table 1 — P-value data of the repeated root measurements from a mixed-effect model with variables genotype, soil, and water treatments (plus their interaction) for each day, plus the combined analysis with all variables combined with the time element (day). The P-values highlighted (green) are those that are classified as significant (P>0.05). [file DataSheet_1.pdf]

Supplementary Table 1) P-value data of the repeated root measurements from a mixed-effect model with variables Cultivar, Top Soil, and Water treatment (plus their interaction) for each day, plus the combined analysis with all variables combined with the time element (day). The P-values highlighted (green) are those that are classified as significant (P>0.05).

Day	Effect	Axial Root Length	Lateral Root Length	Total Root Length	Root System Depth	Root System Width	Convex Hull Area
	Genotype	0.7847	-	0.7847	0.9405	0.0237	0.1343
	TopSoil	0.5953	-	0.5953	0.1384	0.1308	0.1368
	Water	0.0065	-	0.0065	0.0006	0.4498	0.0103
**3**	*Genotype*TopSoil*	0.137	-	0.137	0.0812	0.1371	0.0568
	*Genotype*Water*	0.0454	-	0.0454	0.0523	0.3436	0.101
	*TopSoil*Water*	0.0644	-	0.0644	0.1987	0.0512	0.0304
	*Genoty*TopSoil*Water*	0.4364	-	0.4364	0.9835	0.1185	0.3039
	Genotype	0.2744	-	0.2744	0.7389	0.9888	0.6103
	TopSoil	0.0946	-	0.0946	0.0373	0.018	0.018
	Water	<.0001	-	<.0001	<.0001	0.0666	<.0001
**5**	*Genotype*TopSoil*	0.1425	-	0.1425	0.1798	0.5151	0.0546
	*Genotype*Water*	0.184	-	0.184	0.0937	0.283	0.7256
	*TopSoil*Water*	0.05	-	0.05	0.1661	0.1703	0.9014
	*Genoty*TopSoil*Water*	0.7626	-	0.7626	0.9494	0.1993	0.1229
	Genotype	0.1587	0.8574	0.1859	0.2855	0.2926	0.6636
	TopSoil	0.0725	<.0001	0.0203	0.0213	0.0615	0.0346
	Water	<.0001	<.0001	<.0001	<.0001	0.0407	<.0001
**7**	*Genotype*TopSoil*	0.0645	0.2953	0.0495	0.352	0.6237	0.3952
	*Genotype*Water*	0.5464	0.5166	0.6286	0.0471	0.6169	0.9796
	*TopSoil*Water*	0.0207	<.0001	0.0001	0.0431	0.3998	0.6527
	*Genoty*TopSoil*Water*	0.8658	0.2931	0.7818	0.3837	0.049	0.0312
	Genotype	0.2675	0.2481	0.21	0.0627	0.0516	0.8156
	TopSoil	0.0845	<.0001	0.0023	0.0689	0.0572	0.0261
	Water	<.0001	<.0001	<.0001	<.0001	0.0513	<.0001
**10**	*Genotype*TopSoil*	0.18	0.5294	0.2528	0.6811	0.2728	0.3056
	*Genotype*Water*	0.9119	0.5061	0.7917	0.3758	0.778	0.7095
	*TopSoil*Water*	0.0452	<.0001	<.0001	0.4746	0.7539	0.8391
	*Genoty*TopSoil*Water*	0.8008	0.5165	0.6902	0.2059	0.0154	0.0084
	Genotype	0.1677	0.2236	0.1257	0.012	0.0928	0.9236
	TopSoil	0.0717	<.0001	0.0039	0.0841	0.0152	0.0028
	Water	<.0001	<.0001	<.0001	<.0001	0.1112	<.0001
**12**	*Genotype*TopSoil*	0.2161	0.046	0.1777	0.495	0.1246	0.0719
	*Genotype*Water*	0.9326	0.7791	0.9926	0.502	0.8006	0.7682
	*TopSoil*Water*	0.1234	<.0001	0.005	0.8328	0.6188	0.5125
	*Genoty*TopSoil*Water*	0.7657	0.4405	0.6046	0.1755	0.0078	0.0031
	Genotype	0.1422	0.1389	0.099	0.0071	0.077	0.7448
	TopSoil	0.0297	0.0014	0.002	0.0705	0.0056	0.002
	Water	<.0001	<.0001	<.0001	<.0001	0.3169	<.0001
**14**	*Genotype*TopSoil*	0.3409	0.0594	0.1477	0.5191	0.2446	0.0939
	*Genotype*Water*	0.6915	0.9235	0.7802	0.6817	0.8368	0.989
	*TopSoil*Water*	0.2706	0.0063	0.0513	0.8991	0.6545	0.3769
	*Genoty*TopSoil*Water*	0.855	0.7025	0.7374	0.2585	0.0443	0.0193
	Genotype	0.2194	0.4689	0.2253	0.0023	0.0553	0.6528
	TopSoil	0.0091	0.0062	0.001	0.0426	0.0063	0.0015
	Water	<.0001	0.3215	<.0001	<.0001	0.3843	<.0001
**17**	*Genotype*TopSoil*	0.3637	0.0569	0.1179	0.4395	0.5475	0.101
	*Genotype*Water*	0.6129	0.674	0.8563	0.8231	0.881	0.8933
	*TopSoil*Water*	0.61	0.2051	0.3335	0.6608	0.6846	0.2465
	*Genoty*TopSoil*Water*	0.9278	0.7207	0.7962	0.5934	0.0369	0.0252

19	*Genotype*	0.2653	0.4252	0.2255	0.0028	0.0911	0.4309
	TopSoil	0.0108	0.0033	0.0011	0.0483	0.0181	0.002
	Water	<.0001	0.799	<.0001	<.0001	0.3276	<.0001
	*Genotype*TopSoil*	0.5024	0.0574	0.1467	0.4595	0.3999	0.0603
	*Genotype*Water*	0.6458	0.5207	0.9684	0.6111	0.9191	0.5091
	*TopSoil*Water*	0.7185	0.3364	0.4742	0.6472	0.8418	0.3406
	*Genoty*TopSoil*Water*	0.9354	0.8226	0.8546	0.8471	0.0159	0.0148
21	*Genotype*	0.4961	0.2662	0.2817	0.0413	0.1587	0.3418
	TopSoil	0.0076	0.0007	0.0004	0.0215	0.0137	0.0034
	Water	<.0001	0.131	<.0001	<.0001	0.2241	<.0001
	*Genotype*TopSoil*	0.5767	0.0454	0.1476	0.6695	0.3808	0.0694
	*Genotype*Water*	0.9409	0.6105	0.8468	0.0528	0.6978	0.3112
	*TopSoil*Water*	0.8335	0.5974	0.6614	0.2491	0.9823	0.5563
	*Genoty*TopSoil*Water*	0.9165	0.9046	0.8917	0.9643	0.0168	0.0214
24	*Genotype*	0.6567	0.0273	0.1384	0.0655	0.1318	0.2501
	TopSoil	0.0233	<.0001	0.0003	0.0356	0.0061	0.0019
	Water	<.0001	<.0001	<.0001	<.0001	0.0878	<.0001
	*Genotype*TopSoil*	0.4058	0.0249	0.0733	0.7335	0.3739	0.0944
	*Genotype*Water*	0.7498	0.8266	0.7503	0.0798	0.985	0.1443
	*TopSoil*Water*	0.9149	0.8339	0.96	0.036	0.9755	0.5262
	*Genoty*TopSoil*Water*	0.6925	0.764	0.6692	0.6862	0.0598	0.0723
Total	*t*	<.0001	<.0001	<.0001	<.0001	<.0001	<.0001
	*t*Genotype*	0.198	0.2541	0.14	0.0023	0.0542	0.5215
	*t*TopSoil*	<.0001	<.0001	<.0001	<.0001	<.0001	<.0001
	*t*Water*	<.0001	<.0001	<.0001	<.0001	0.0004	<.0001
	*t*Genotype*TopSoil*	0.0005	0.2027	0.0002	0.0077	0.0069	0.0013
	*t*Genotype*Water*	0.9721	0.941	0.6222	0.0021	0.7077	0.3651
	*t*TopSoil*Water*	<.0001	<.0001	<.0001	0.2897	0.4521	0.0719
	*t*Genot*TopSoi*Water*	0.3315	0.2792	0.1857	0.0262	<.0001	<.0001

Supplementary Table 2) P-value data of the repeated shoot measurements from a mixed-effect model with variables Cultivar, Top Soil, and Water treatment (plus their interaction) for each day, plus the combined analysis with all variables combined with the time element (day) for those with more than two time points. The P-values highlighted (green) are those that are classified as significant (P>0.05).

Day	Effect	Height	GS	SPAD	Fv/Fm	Mn predict	P predict
7	*Genotype*	0.4729	0.3832	-	-	-	-
	TopSoil	<.0001	0.1436	-	-	-	-
	Water	<.0001	<.0001	-	-	-	-
	*Genotype*TopSoil*	0.0991	0.1876	-	-	-	-
	*Genotype*Water*	0.5677	0.3147	-	-	-	-
	*TopSoil*Water*	0.004	0.1158	-	-	-	-
	*Genoty*TopSoil*Water*	0.8612	0.2568	-	-	-	-
10	*Genotype*	-	0.2502	0.0253	-	-	-
	TopSoil	-	0.0013	0.0287	-	-	-
	Water	-	0.0002	<.0001	-	-	-
	*Genotype*TopSoil*	-	0.9458	0.201	-	-	-
	*Genotype*Water*	-	0.8114	0.1008	-	-	-
	*TopSoil*Water*	-	0.3657	0.0102	-	-	-
	*Genoty*TopSoil*Water*	-	0.9498	0.1448	-	-	-
14	*Genotype*	-	0.7507	0.1845	-	-	-
	TopSoil	-	0.0315	0.0139	-	-	-
	Water	-	<.0001	0.0503	-	-	-
	*Genotype*TopSoil*	-	0.6539	0.0145	-	-	-
	*Genotype*Water*	-	0.7507	0.2516	-	-	-
	*TopSoil*Water*	-	0.2798	0.2682	-	-	-
	*Genoty*TopSoil*Water*	-	0.7766	0.3859	-	-	-
17	*Genotype*	-	0.342	0.8294	0.0106	0.0007	0.0261
	TopSoil	-	0.022	0.0531	0.1549	0.0231	0.7887
	Water	-	<.0001	<.0001	<.0001	<.0001	<.0001
	*Genotype*TopSoil*	-	0.0984	0.0001	0.483	0.3752	0.2698
	*Genotype*Water*	-	0.2863	0.4486	0.8194	0.7728	0.9371
	*TopSoil*Water*	-	0.1109	0.165	0.9841	0.8593	0.1435
	*Genoty*TopSoil*Water*	-	0.291	0.3876	0.2374	0.333	0.1205
21	*Genotype*	0.0016	0.8569	0.4235	0.5136	0.7245	0.8518
	TopSoil	0.1522	0.0061	0.0561	0.5821	0.8239	0.4323
	Water	<.0001	<.0001	0.0002	<.0001	<.0001	<.0001
	*Genotype*TopSoil*	0.5762	0.7136	0.3172	0.4132	0.5517	0.043
	*Genotype*Water*	0.3457	0.8569	0.8544	0.5975	0.8764	0.4754
	*TopSoil*Water*	0.0164	0.0037	0.804	0.5612	0.0518	0.3807
	*Genoty*TopSoil*Water*	0.2439	0.9829	0.0803	0.6563	0.0507	0.6905
24	*Genotype*	<.0001	0.4252	0.4313	-	-	-
	TopSoil	0.004	0.3585	0.2681	-	-	-
	Water	<.0001	<.0001	<.0001	-	-	-
	*Genotype*TopSoil*	0.4013	0.6494	0.0616	-	-	-
	*Genotype*Water*	0.6075	0.273	0.4588	-	-	-
	*TopSoil*Water*	0.7266	0.24	0.4528	-	-	-
	*Genoty*TopSoil*Water*	0.612	0.8873	0.6467	-	-	-
Total	*t*	<.0001	<.0001	<.0001	-	-	-
	*t*Genotype*	0.0017	0.5449	0.4678	-	-	-
	*t*TopSoil*	0.0001	0.0025	0.028	-	-	-
	*t*Water*	<.0001	<.0001	<.0001	-	-	-
	*t*Genotype*TopSoil*	0.1811	0.4777	0.1538	-	-	-
	*t*Genotype*Water*	0.2982	0.6492	0.7866	-	-	-
	*t*TopSoil*Water*	0.0297	0.0097	0.9359	-	-	-
	*t*Genot*TopSoi*Water*	0.2215	0.9726	0.1189	-	-	-

Supplementary Table 3) P-value data of the destructive shoot and root measurements from a mixed-effect model with variables Cultivar, Top Soil, and Water treatment (plus their interaction) for the last (24th) day of the experiment. The P-values highlighted (green) are those that are classified as significant (P>0.05).

Effect	Leaf Area	Shoot FW	Shoot DW	Log Shoot FW:DW Ratio
Genotype	0.666	0.7886	0.4349	0.1615
TopSoil	0.0006	0.0002	0.0002	0.3843
Water	<.0001	<.0001	<.0001	<.0001
*Genotype*TopSoil*	0.0063	0.0151	0.01	0.2316
*Genotype*Water*	0.4669	0.3576	0.4557	0.5327
*TopSoil*Water*	0.6181	0.4093	0.8309	0.5262
*Genoty*TopSoil*Water*	0.2069	0.3257	0.1753	0.059

Effect	Root DW	Log Root:Shoot Ratio	Nitrogen Concentration	Nitrogen per Plant
Genotype	0.5311	0.0724	0.5362	0.3585
TopSoil	0.594	0.014	0.0993	0.006
Water	<.0001	0.0002	0.0005	<.0001
*Genotype*TopSoil*	0.3295	0.8738	0.8441	0.0006
*Genotype*Water*	0.5924	0.397	0.945	0.977
*TopSoil*Water*	0.7702	0.3339	0.2777	0.0265
*Genoty*TopSoil*Water*	0.992	0.5434	0.6763	0.0037

Supplementary Table 4) P-value data of the root length measurements from a mixed-effect model with variables Cultivar, Top Soil, and Water treatment (plus their interaction) for the last (24th) day of the experiment, divided into either three horizontal (**A**) or four vertical (**B**) sections. The Centre and Topsoil sections of the Horizontal and Vertical sectioning, respectively, contain the treated soil sections. The P-values highlighted (green) are those that are classified as significant (P>0.05).

A) Horizontal Sections

	Effect	Axial Root Length	Lateral Root Length
**Left**	*Genotype*	0.8748	0.0569
	TopSoil	0.7294	0.0285
	*Genotype*TopSoil*	0.8129	0.0304
	Water	<.0001	0.7512
	*Genotype*Water*	0.8717	0.9335
	*TopSoil*Water*	0.7558	0.6259
	*Genoty*TopSoil*Water*	0.0741	0.3175
**Centre**	*Genotype*	0.2855	0.4031
	TopSoil	0.6349	0.0359
	*Genotype*TopSoil*	0.2829	0.268
	Water	<.0001	<.0001
	*Genotype*Water*	0.519	0.7798
	*TopSoil*Water*	0.6754	0.3649
	*Genoty*TopSoil*Water*	0.2553	0.0362
**Right**	*Genotype*	0.0997	0.2669
	TopSoil	0.4612	0.0716
	*Genotype*TopSoil*	0.3859	0.5726
	Water	0.3396	0.7237
	*Genotype*Water*	0.1814	0.8336
	*TopSoil*Water*	0.4012	0.568
	*Genoty*TopSoil*Water*	0.8664	0.9962

B) Vertical Sections

	Effect	Axial Root Length	Lateral Root Length
**Topsoil**	*Genotype*	0.6974	0.4764
	TopSoil	0.0567	<.0001
	*Genotype*TopSoil*	0.1257	0.1858
	Water	<.0001	<.0001
	*Genotype*Water*	0.4164	0.7156
	*TopSoil*Water*	0.8655	<.0001
	*Genoty*TopSoil*Water*	0.1461	0.5307
**Top**	*Genotype*	0.8611	0.3624
	TopSoil	0.9452	0.1928
	*Genotype*TopSoil*	0.4624	0.4176
	Water	<.0001	0.2499
	*Genotype*Water*	0.0009	0.4626
	*TopSoil*Water*	0.5407	0.0962
	*Genoty*TopSoil*Water*	0.2405	0.2302
**Middle**	*Genotype*	0.4014	0.1898
	TopSoil	0.0068	0.0014
	*Genotype*TopSoil*	0.7396	0.1913
	Water	<.0001	<.0001
	*Genotype*Water*	0.467	0.954
	*TopSoil*Water*	0.6242	0.1445
	*Genoty*TopSoil*Water*	0.9316	0.4983
**Bottom**	*Genotype*	0.7526	0.102
	TopSoil	0.0806	0.1353
	*Genotype*TopSoil*	0.6162	0.2693
	Water	<.0001	<.0001
	*Genotype*Water*	0.0586	0.1315
	*TopSoil*Water*	0.6263	0.1634
	*Genoty*TopSoil*Water*	0.6929	0.3126
